# Transcriptional Profiling Identifies Location-Specific and Breed-Specific Differentially Expressed Genes in Embryonic Myogenesis in Anas Platyrhynchos

**DOI:** 10.1371/journal.pone.0143378

**Published:** 2015-12-02

**Authors:** Rong-Ping Zhang, He-He Liu, Jun-Ying Liu, Ji-Wei Hu, Xi-Ping Yan, Ding-Min-Cheng Wang, Liang Li, Ji-Wen Wang

**Affiliations:** Farm Animal Genetic Resources Exploration and Innovation Key Laboratory of Sichuan Province, Sichuan Agricultural University, Ya’an 625014, China; Wageningen UR Livestock Research, NETHERLANDS

## Abstract

Skeletal muscle growth and development are highly orchestrated processes involving significant changes in gene expressions. Differences in the location-specific and breed-specific genes and pathways involved have important implications for meat productions and meat quality. Here, RNA-Seq was performed to identify differences in the muscle deposition between two muscle locations and two duck breeds for functional genomics studies. To achieve those goals, skeletal muscle samples were collected from the leg muscle (LM) and the pectoral muscle (PM) of two genetically different duck breeds, Heiwu duck (H) and Peking duck (P), at embryonic 15 days. Functional genomics studies were performed in two experiments: Experiment 1 directly compared the location-specific genes between PM and LM, and Experiment 2 compared the two breeds (H and P) at the same developmental stage (embryonic 15 days). Almost 13 million clean reads were generated using Illumina technology (Novogene, Beijing, China) on each library, and more than 70% of the reads mapped to the Peking duck (Anas platyrhynchos) genome. A total of 168 genes were differentially expressed between the two locations analyzed in Experiment 1, whereas only 8 genes were differentially expressed when comparing the same location between two breeds in Experiment 2. Gene Ontology (GO) and the Kyoto Encyclopedia of Genes and Genomes pathways (KEGG) were used to functionally annotate DEGs (differentially expression genes). The DEGs identified in Experiment 1 were mainly involved in focal adhesion, the PI3K-Akt signaling pathway and ECM-receptor interaction pathways (corrected P-value<0.05). In Experiment 2, the DEGs were associated with only the ribosome signaling pathway (corrected P-value<0.05). In addition, quantitative real-time PCR was used to confirm 15 of the differentially expressed genes originally detected by RNA-Seq. A comparative transcript analysis of the leg and pectoral muscles of two duck breeds not only improves our understanding of the location-specific and breed-specific genes and pathways but also provides some candidate molecular targets for increasing muscle products and meat quality by genetic control.

## Introduction

Myogenesis is a highly complex physiology process that involves myogenic progenitor proliferation, myoblast proliferation and differentiation, and the formation of none-nuclei and multi-nuclei myotubes and eventually mature muscle. As such, myogenesis is highly regulated by numerous signature pathways and genes [1]. Embryo myogenesis is pivotal for muscle production in adult livestock because the myofiber number is determined during the embryonic stage for most animals and does not increase in the postnatal period. Rather muscle mass gain in adult livestock mainly depends on increasing the length and thickness of existing myofibers, a process referred to as hypertrophy [2]. In addition, muscle stem (satellite) cells also play a vital role in muscle development in adults, specifically in muscle regeneration during muscle injury, overload [3] and exercise[4, 5]. Satellite cells originate from somites in the embryo stage and reside between the basement membrane and the myofiber sarcolemma in adults [6]. Unfortunately, the number and function of satellite cells will inevitably continually decrease with age [7]. Therefore, the number of myofibers established in the embryo stage is the critical determinant of muscle production in livestock. A previous study using microarray hybridization reported that, in turkeys, a higher number of differentially expressed genes occurred early in development (day 18 of the embryonic stage) than at 1 day and 16 weeks after birth, suggesting that the phenotypic differences in adults between the two turkey lines may largely be determined during embryonic myogenesis [8]. Other studies have demonstrated that muscle growth is predominantly determined during prenatal skeletal muscle development [9, 10]. Thus, evidence indicates that the embryonic stage is an important period in the research of muscle development, and a better understanding of the genes and pathways involved is necessary.

Waterfowl breeding plays an important and unique role in agricultural development. However, compared to human, mouse or chicken, studies of the myogenesis mechanisms in duck, a non-model species, are incomplete and are still in their infancy. Most of these studies in duck have mainly focused on comprehensively investigating the expression patterns of a few crucial regulative genes. The key genes six1 [11], Pax3/7 [12], MRFs (MyoD, MyoG and MRF4) [12, 13], mTOR and S6k [14] regulate myoblast proliferation and myofiber hypertrophy. These studies identified expression differences of such genes between pectoral and leg muscles in Peking duck have been identified. In addition, previous studies have shown that the carcass and meat quality of duck are influenced by breed and sex [15, 16]. These results indicate that the numerous biological and genetic differences between skeletal muscles depend on their anatomical location and breed.

**Fig 1:**
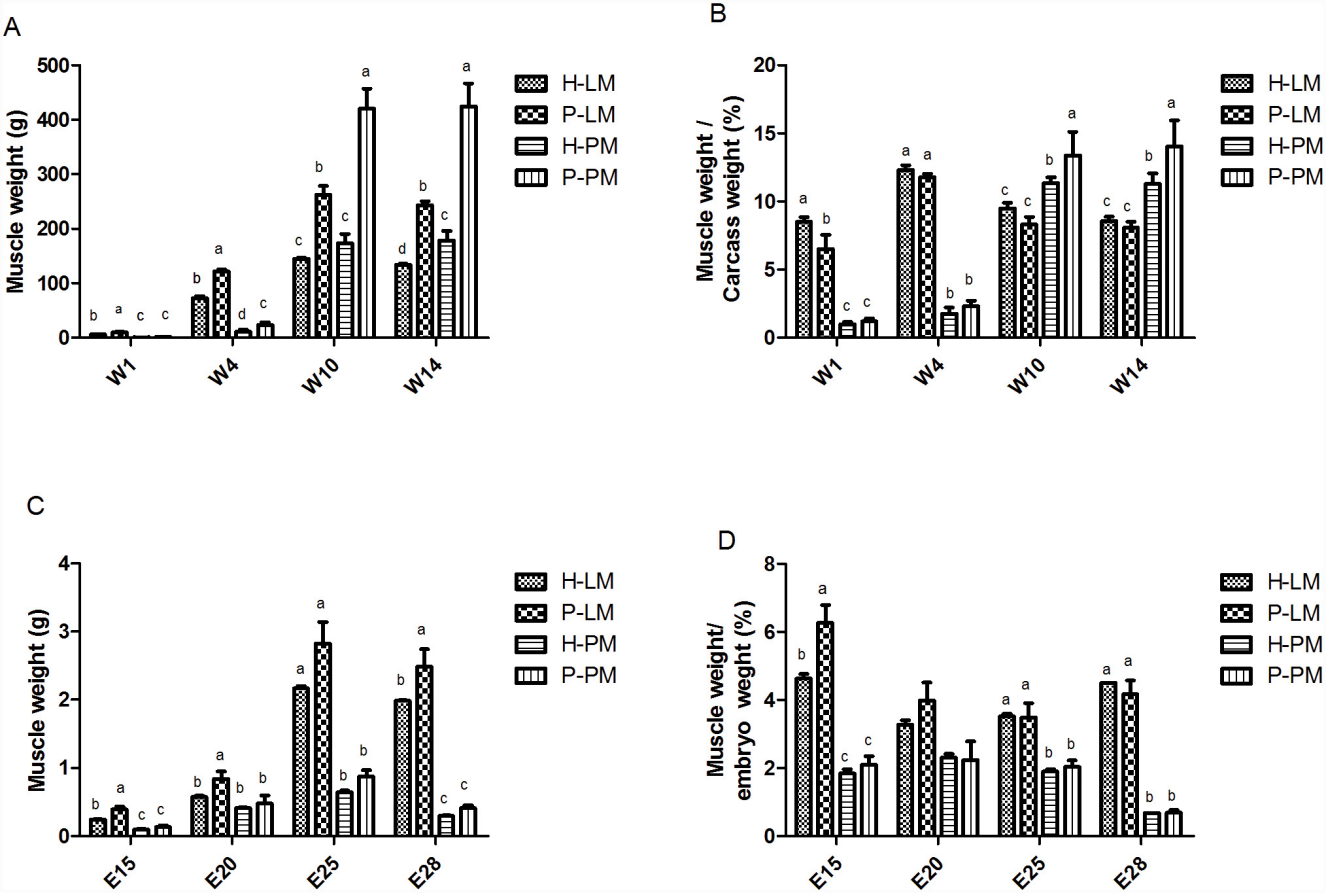
Skeletal muscle variations in leg muscle (LM) and pectoral muscle (PM) between Heiwu duck (H) and Peking duck (P) during development. A. The increase in the weight of four skeletal muscles from post-hatching week 1 (W1) to week 16 (W14). B. The changing rate of skeletal muscle weight to carcass weight from post-hatching week 1 (W1) to week 16 (W14). C. The variation in the weight of the four skeletal muscles in embryo from embryonic day 15 (E15) to day 28 (E28). D. The changing rate of skeletal muscle weight to carcass weight from embryonic day 15 (E15) to day 28 (E28). At each time point (W1, W4, W10, etc), statistically significant differences are indicated by different letters (a, b, c, etc). At a given time point, any samples that are not significantly different are labeled with the same letter.

In this study, we used several individuals from two native duck breeds, Heiwu duck and Peking duck, to identify changes in gene expression which may be responsible for the differences in muscle development between locations and breeds. We observed phenotypic differences between pectoral and leg muscle and between the same muscle type from both breeds (for detailed data, see Fig 1). To further investigate these differences, a highly effective and accurate digital gene expression (DGE) technology was used to obtain abundant sequences at the transcript level. In 2013, Huang et al. released the draft genome sequence of Anas Platyrhynchos acquired using Illumine technology [17], which equips us to better study myogenesis of duck using DGE technology. This study will help us to identify differentially expressed genes related to pectoral muscle and leg muscle myogenesis in two duck breeds. While our data was collected from a relatively small sample set, this work will likely still be helpful for understanding the molecular basis of the different muscle development capabilities of the leg and pectoral muscle of Peking duck and Heiwu duck, providing further knowledge and new clues for investigation of muscle development mechanisms, and improving duck breeding research.

## Materials and Methods

### Ethics Statement

All ducks were obtained from the Sichuan Agriculture University Waterfowl Breeding Experimental Farm, Sichuan, China. This study was carried out according to Beijing Animal Welfare Committee (Beijing, China) and approved by the institutional Animal Care and Use Committee of Sichuan Agriculture University (Permit Number: DKY B20121405). All surgery was performed under sodium pentobarbital anesthesia, and all efforts were made to minimize suffering.

### Animal materials

Peking duck (Anas platyrhynchos domestica) (average weight 80 g) and Heiwu duck (average weight 75 g) eggs were incubated under the same conditions, 37±0.5°C and 86–87% humidity. After incubation for 15 days at the Sichuan Agriculture University Waterfowl Breeding Experimental Farm, leg muscles (LM) and pectoral muscles (PM) were collected from the Heiwu duck (H) and Peking duck (P) specimens, separately, five individual ducks were used per breed. All of the muscles were frozen in liquid nitrogen and stored at -80°C prior to examination.

During the embryo stage, LM and PM of the H and P specimens were collected at 15, 20, 25 and 28 days of incubation, five individual ducks were used per breed at each time point. All of the samples were weighed immediately after isolation from the embryo.

Post-hatching, the duck breeds were raised under the same conditions. For both breeds, six ducks (three male and three female) were weighed and slaughtered at every week until 16 weeks. Then, the LM and PM of six ducks were isolated and weighed separately. These procedures were approved by the Beijing Animal Welfare Committee (Beijing, China).

### RNA preparation, Illumina RNA-sequencing

The total RNA from four group muscles (H-LM, H-PM, P-LM and P-PM) was extracted using Trizol reagent (Takara, China) according to the manufacturer’s instructions. Five individuals’ RNA samples per group were equally mixed to generate an RNA pool. The RNA purity was checked using the NanoPhotometer^®^ spectrophotometer (IMPLEN, CA, USA). The RNA integrity was assessed using the RNA Nano 6000 Assay Kit of the Bioanalyzer 2100 system (Agilent Technologies, CA, USA).

Sequencing libraries were generated using the NEBNext^®^ Ultra^™^ RNA Library Prep Kit for Illumina^®^ (NEB, USA) following the manufacturer’s recommendations, and index codes were added to attribute sequences to each sample. Briefly, mRNA was purified from the total RNA using poly-T oligo-attached magnetic beads. Fragmentation was carried out using divalent captions under elevated temperature in the NEBNext First Strand Synthesis Reaction Buffer (5×). First-strand cDNA was synthesized using a random hexamer primer and M-MuLV Reverse Transcriptase (RNase H-). Second-strand cDNA synthesis was subsequently performed using DNA Polymerase I and RNase H. The remaining overhangs were converted into blunt ends via exonuclease/polymerase activities. After the adenylation of the 3’ ends of DNA fragments, NEBNext Adaptor with a hairpin loop structure was ligated to prepare for hybridization. To preferentially select cDNA fragments of 150~200 bp in length, the library fragments were purified with the AMPure XP system (Beckman Coulter, Beverly, USA). Then, 3 μl USER Enzyme (NEB, USA) was used with size-selected, adaptor-ligated cDNA at 37°C for 15 min followed by 5 min at 95°C before PCR. Then, PCR was performed with Phusion High-Fidelity DNA polymerase, Universal PCR primers and Index (X) Primer. Finally, the PCR products were purified (AMPure XP system) and the library quality was assessed on the Agilent Bioanalyzer 2100 system.

### DGE read annotation

To identify the gene expression patterns in the skeletal muscle of the Peking duck and Heiwu duck specimens, all of the clean reads were annotated by mapping to the sequenced genome of Anas [17] using the TopHat v2.0.9 software. For gene expression analysis, HTSeq v0.5.4p3 was used to count the read numbers that were mapped to each gene. Then, RPKM (Reads Per Kilo bases per Million reads) was used to calculate and normalize the number of expression tags. A DEG analysis of two locations/breeds was performed using the DESeq R package (1.10.1). DESeq provides statistical routines for determining the differential expression in digital gene expression data using a model that was based on the negative binomial distribution. Genes with an adjusted P-value < 0.05 found by DESeq were assigned as differentially expressed. The Gene Ontology (GO) enrichment analysis of differentially expressed genes was implemented by the GOseq R package, in which gene length bias was corrected. GO terms with a corrected P-value < 0.05 were considered significantly enriched by differentially expressed genes. We used KOBAS software to test the statistical enrichment of differentially expressed genes in KEGG pathways. All of the sequence data were submitted to the GEO database, and the GEO accession number is GSE65628.

### Quantitative real-time PCR confirmation

A total of 15 genes were chosen randomly and detected by quantitative real-time PCR (RT-PCR) to confirm the accurate DGE. All primers (S1 Table) were designed using Primer 5.0 software and synthesized by BGI Company (China). Sample RNAs (1 μg) were reverse-transcribed to cDNA using a reverse-transcription system (Takara, Dalian, China), three individuals’ RNA were used per group at E15, and RT-PCR for each sample was conducted in triplicate. The reaction was run using the IQTM5 System (Bio-Rad, Hercules, CA), and the data were analyzed by the 2^-ΔΔCt^ method using β-actin and GAPDH as internal reference genes. A statistical analysis was performed with GLM processes and t-test using SAS 8.0 software (SAS Institute Inc., Cary, NC).

## Results

### Phenotypic analysis of skeletal muscle in ducks

In order to identify breed-specific mechanisms that may contribute to the differences in muscle development capability, two phenotypically different duck breeds were examined. We began by comparing two different muscle groups within each breed. As shown in Experiment 1 (Fig 1A), in both breeds skeletal muscle has a significant weight difference between LM and PM, at all measurement time points except W10 in the Heiwu duck. To take into account the carcass weight of each duck, we calculated the ratio of LM and PM weight to carcass weight and found that every ratio was significantly different between locations, and this was true of both breeds (Fig 1B). When comparing across breeds in Experiment 2, we found that Peking duck has a higher skeletal muscle weight than Heiwu duck in the postnatal period. However, the ratio of LM weight to carcass weight was significantly different between the duck breeds only at W1, while the ratio of PM weight to carcass weight differed significantly at W10 and W14 (Fig 1B). These data indicate that Peking duck and Heiwu duck are two phenotypically extreme duck breeds, and these differences are more obvious in PM than in LM.

We then went on to examine skeletal development in embryos. In Experiment 1, the muscle weight was significantly different between locations for both breeds during the embryo period, with the exception of the Heiwu duck at E20 (Fig 1C). Comparing across breeds in Experiment 2, LM weight, unlike PM weight, was significantly different between the Peking duck and the Heiwu duck, except at E25. However, the ratio of LM or PM to embryo weight was not significantly different between the Peking duck and the Heiwu duck, with a sole exception. The only ratio to show a significant difference between the breeds was the LM:embryo weight ratio at E15 (Fig 1D). These weight data from both either in the embryo or at and the post-hatching periods indicate phenotypic differences between muscle locations and breeds.

### Analysis and alignment of the digital gene expression (DGE) profile

In this study, four DGE-read libraries (H-LM, H-PM, P-LM and P-PM) were constructed using LM and PM tissues from two phenotypically extreme duck breeds (H and P). The statistics of the DGE reads are shown in Table 1. More than 13 million raw reads were generated for each library. After filtering the adapter reads, more than 10% of the N (uncertain base information) reads and low-quality reads and more than 97% of the raw reads were clean reads for each library. On average, 70% of the transcripts mapped to Peking duck (Anas platyrhynchos) genome.

**Table 1:** Categorization and abundance of reads.

Summary	H-LM	H-PM	P-LM	P-PM
Raw reads	17111295	13082066	14496812	14435361
Clean reads	16624232	12721325	14098020	14037879
Error rate (%)	0.04	0.04	0.04	0.04
Mapped reads	11659490	9065390	10065643	10070140
% mapped	70.14%	71.26%	71.4%	71.74%

### Analysis of the level of gene expression

**Fig 2:**
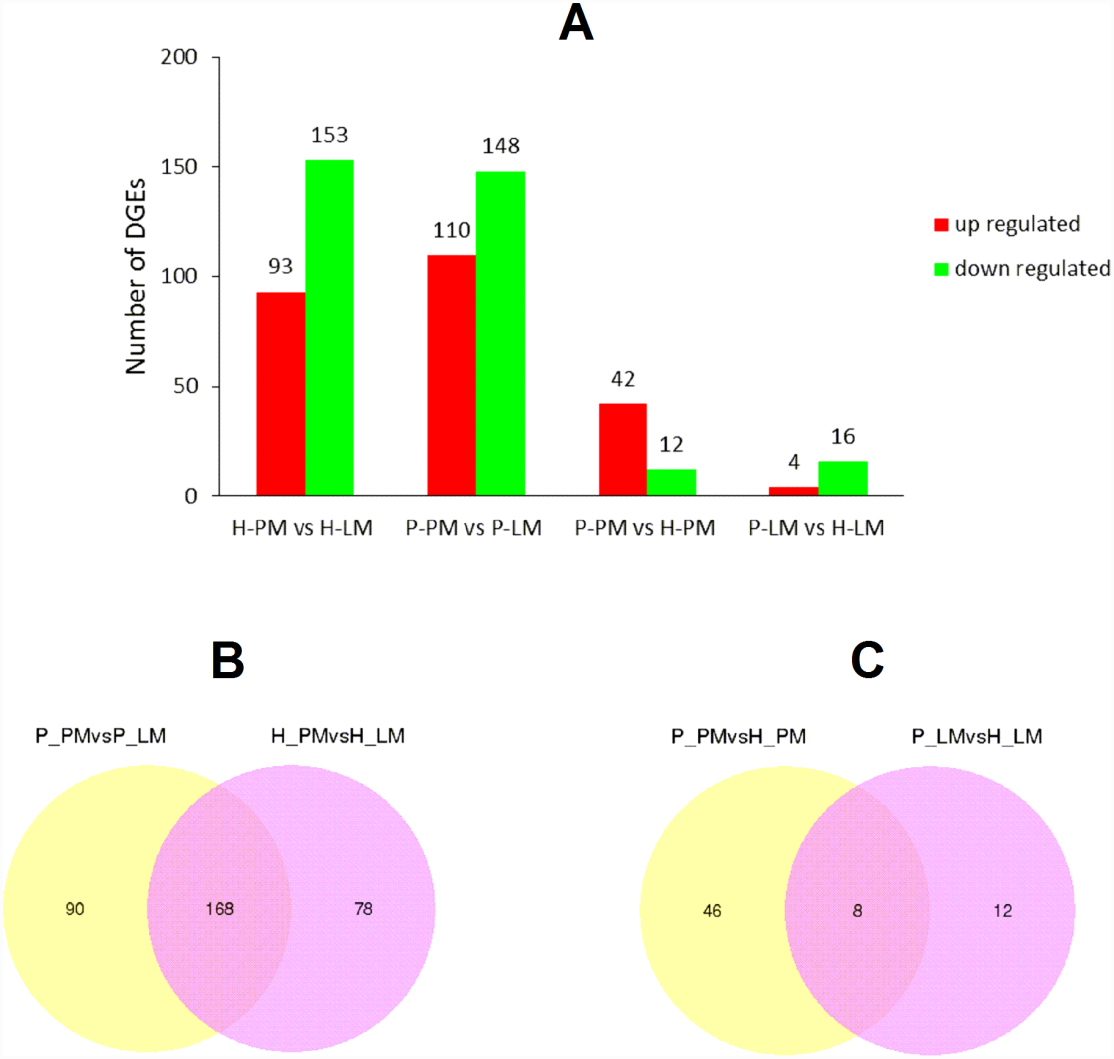
The distribution of DEGs in each library. A: The numbers of differentially expressed genes in each comparison. The up-regulated genes are shown in red, whereas the down-regulated genes are shown in green. “A” was the control group, and “B” was the experimental group in “A vs B”. B. The distribution of DEGs that were found to be commonly and specifically expressed between locations in Experiment 1. C. The distribution of DEGs that were found to be commonly and specifically expressed between breeds in Experiment 2.

To identify genes that were differentially expressed in the muscle tissues of the duck breeds, we compared pairs of DGE profiles in the four libraries (Experiment 1: H-PM vs H-LM and P-PM vs P-LM, Experiment 2: P-PM vs H-PM and P-LM vs H-LM) to analyze gene expression variations. |log.2fold-change|>0.5 and P-value <0.05 were used as the thresholds for significant differential gene expression (Fig 2A). In Experiment 1, a total of 336 genes were differentially expressed between LM and PM. Of these, 246 genes were significantly affected in the H breed, and 258 genes were significantly affected in the P breed (Fig 2B). Many fewer genes were found to be significantly different between breeds in Experiment 2. Only 8 genes were differentially expressed between the two duck breeds (Fig 2C). All of the DEGs are listed in the S1 Excel file. As expected, the majority of gene expression changes occurred between LM and PM, and these data indicate that muscle location rather than breed differentiation is mainly responsible for muscle disposition differences (S1 Fig).

### Validation of DEGs by RT-PCR

**Fig 3:**
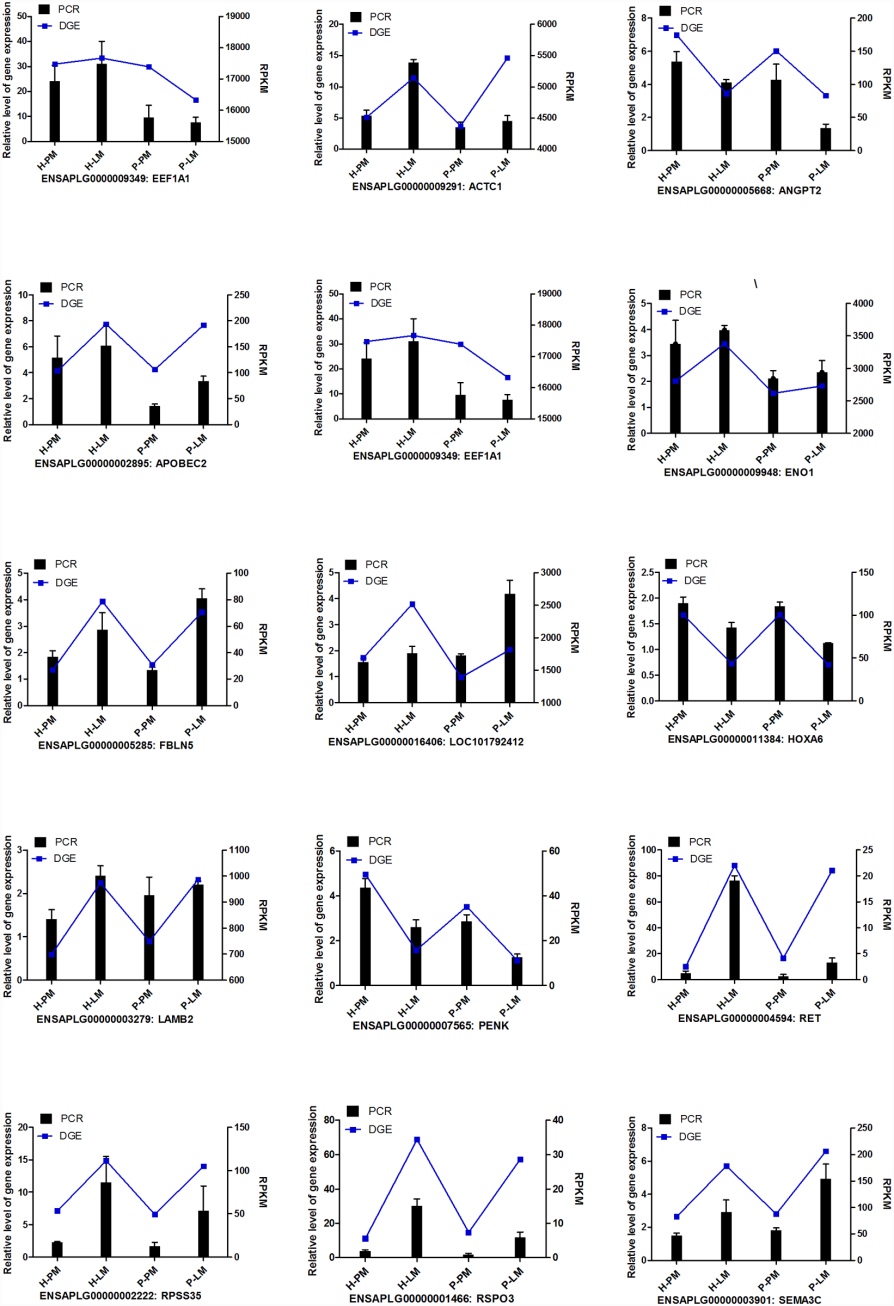
RT-PCR validates the correction of RNA-Seq. The relative expression levels were calculated using β-actin and GAPDH as the internal controls. RPKM: the number of reads per kilo bases per million reads (RPKM).

To confirm the differentially expressed genes from the RNA-Seq data, 15 genes (ACTA1, ACTC1, ANGPT2, APOBEC0, EEF1A1, ENO1, FBLN5, LOC101792412, HOXA6, LAMB2, PENK, RET, RPSS35, RSPO3, and SEMA3C) were chosen randomly and measured by real-time PCR. Only EEFIA1 and LOC101792412 were common between Experiment 1 and Experiment 2. The RT-PCR expression profiles of the fifteen genes closely resemble the expression pattern obtained from the DGE results (Fig 3), except that some genes have a different relative expression level on DGE at a specific muscle tissue. The RT-PCR expression profiles therefore indicate the reliability of the RNA-Seq data.

### DEGs involved in myogenesis and muscle metabolism

Studies have showed that many genes are involved in myogenesis and muscle metabolism. Some key genes related to skeletal muscle development are listed in Table 2. Our data reveal that two myosin heavy-chain isoforms (MYH7b and MYH15) and three myosin light-chain isoforms (MYL1, MYL3 and MYL10) were highly expressed in either LM or PM with the same breed. However, only MYH7b was common between Experiment 1 and Experiment 2 and was up-regulated in the P breed compared to the H breed. We also observed that some important genes that have been implicated in collagen fibril organization and biosynthetic processes, such as COL11A1, COL12A1, COL14A1, COL1A2, COL5A2 and COL9A1, were down-regulated in PM compared to LM. Of troponins and tropomyosins, TNNI2, TNNT3, TPM2 and TPM3 exhibited similar profiles with high expression in LM. Additionally, angiopoietin 2 (ANGPT2) and platelet-derived growth factor (PDGH), which are involved in the PI3K/AKT signaling pathway, were up-regulated in PM. In Experiment 2, DEGs were mainly concentrated in ribosome proteins (RPS6, RPS23, RPL36, etc.).

**Table 2:** Some key different expression genes in myogenesis.

Gene Description	Gene Symbol	log2.Fold-change	q-value	log2.Fold-change	q-value	GeneBank ID
In experiment 1		H-PM VS H-LM		P-PM VS P-LM		
angiopoietin 2	ANGPT2	1.0141	1.81E-06	0.85283	0.000386	NW_004676321.1
collagen type XI alpha 1	COL11A1	-1.3764	6.91E-25	-0.86086	3.15E-10	NW_004678344.1
collagen type XII alpha 1	COL12A1	-0.72494	6.77E-26	-0.44991	5.60E-09	NW_004678503.1
collagen type XIV alpha 1	COL14A1	-1.4077	2.66E-72	-1.3223	6.88E-65	NW_004677473.1
collagen type I alpha 2	COL1A2	-1.0238	0	-0.96764	0	NW_004676428.1
collagen type V alpha 2	COL5A2	-0.6992	1.14E-38	-0.49349	8.29E-20	NW_004676457.1
collagen type IX alpha 1	COL9A1	-3.0763	1.08E-05	-2.5744	0.000195	NW_004676356.1
catenin (cadherin-associated protein) beta 1 88kDa	CTNNB1	0.15351	0.014254	0.22862	1.17E-05	NW_004676826.1
fibrillin 1	FBN1	-0.98987	8.26E-47	-1.4004	1.18E-10	NW_004677012.1
fibronectin 1	FN1	-0.61995	1.19E-49	-0.43585	1.75E-24	NW_004677206.1
laminin alpha 2	LAMA2	-0.5056	0.0009325	-0.48159	0.001914	NW_004676361.1
laminin gamma 1 (formerly LAMB2)	LAMC1	-0.4805	8.27E-08	-0.39883	2.74E-05	NW_004676361.1
myosin binding protein C slow type	MYBPC1	-0.88953	5.39E-40	-0.68899	3.10E-63	NW_004677175.1
myosin heavy chain 15	MYH15	-0.7855	0.0012092	-1.0649	1.43E-55	NO
myosin heavy chain 7B cardiac muscle beta	MYH7B	0.27897	3.23E-06	0.55354	5.52E-05	NW_004676474.1
myosin light chain 1 alkali; skeletal fast	MYL1	-0.56895	5.75E-42	-1.2408	3.46E-17	NO
myosin light chain 10 regulatory	MYL10	-1.3777	1.61E-78	-1.1201	3.10E-63	NO
myosin light chain 3 alkali; ventricular skeletal slow	MYL3	-1.8743	2.79E-40	-1.5084	1.81E-30	NO
platelet derived growth factor C	PDGFC	0.79091	0.032477	0.87484	0.002869	NW_004676768.1
platelet derived growth factor D	PDGFD	0.654	4.22E-07	0.58563	6.43E-06	NW_004676396.1
paired-like homeodomain 1	PITX1	-4.7263	0.016084	-6.5449	0.005697	NW_004676932.1
T-box 4	TBX4	-5.0117	0.0033295	-5.1144	0.001713	NW_004676627.1
troponin I type 2 (skeletal fast)	TNNI2	-0.51741	1.68E-10	-0.43453	5.48E-07	NW_004677025.1
troponin T type 3 (skeletal fast)	TNNT3	-0.52011	7.13E-20	-0.62634	1.80E-27	NO
tropomyosin 2 (beta)	TPM2	-1.1299	1.78E-16	-0.87145	8.30E-11	NO
tropomyosin 3	TPM3	-1.1068	3.08E-24	-0.88126	1.03E-16	NW_004679752.1
Vitronectin	VN			2.5132	2.45E-08	NW_004677611.1
In experiment 2		P-LM vs H-LM		P-PM vs H-PM		
myosin heavy chain 7B cardiac muscle beta	MYH7B	0.27897	3.23E-06	0.55354	5.52E-05	NW_004676474.1
ribosomal protein S6	RPS6	-0.42312	2.117E-16	-0.3713	1.67E-13	NW_004676570.1
ribosomal protein S23	RPS23	-0.46496	5.876E-10	-0.42509	4.90E-10	NW_004676406.1
60S ribosomal protein L37	RPL37	-0.33357	0.0012533	-0.34194	8.10E-05	NW_004677806.1

NO: there is not sequence number for the gene in NCBI.

### Functional and pathway analysis of DEGs

**Fig 4:**
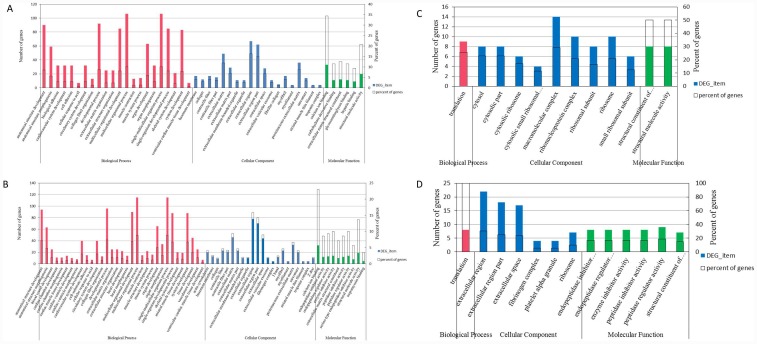
Histogram presents gene ontology classification. The left axis indicates the percentage of the specific category of genes in the main category. The right axis indicates the number of genes in a category. A, B, C and D represent the H-PM vs H-LM.GO, P-PM vs P-LM.GO, P-LM vs H-LM.GO, and P-PM vs H-PM.GO, respectively.

After identifying all of the genes differentially expressed between the two breeds, we annotated the sequences using the Gene Ontology (GO) database (http://www.geneontology.org/) to investigate changes in the patterns of gene expression between locations and breeds. In the H-PM vs H-LM library, 1563 sequences could be classified into 47 secondary level categories (correct P-value <0.001) (Fig 4A). In the P-PM vs P-LM library, 1927 sequences could be categorized into 60 functional groups (Fig 4B). In Experiment 1, “Multicellular organismal process” and “single-multicellular organism process”, “extracellular region” and “calcium ion binding” were found to be dominant in the three corresponding categories. This experiment demonstrated that skeletal muscle location differences are conserved between the Peking duck breed and the Heiwu duck breed. On the other hand, in Experiment 2, 99 and 128 sequences were classified into 12 and 13 secondary functional categories, respectively (Fig 4C and 4D). Only the category “translation” was significantly classified into “biological process” (correct p-value <0.001). The GO analysis showed that the DEGs identified in this study span a broad range of functions and cellular processes.of the identified genes are involved in various processes.

The KEGG data were used to better identify the active biological pathways in the DGE libraries; only significantly enriched genes were displayed in this study (P-value <0.05). In the H-LM vs H-PM libraries, differentially expressed genes were significantly assigned to 10 pathways (Table 3). A total of 11 pathways were significantly enriched in the P-LM vs P-PM library. In Experiment 1, Focal adhesion, the PI3K-Akt signaling pathway and ECM-receptor interaction are the top three pathways. Comparing across breeds in Experiment 2, however, only the ribosome pathway was detected as having differentially expressed components in both muscle locations (Table 3).

**Table 3:** The significant pathways (P<0.05) from KEGG pathway analysis of DEGs.

Pathway item	ID	Sample number	Background number	P-Value	Corrected P-Value
**H-PM VS H-LM**					
Focal adhesion	mmu04510	26	206	4.44E-16	5.91E-14
PI3K-Akt signaling pathway	mmu04151	25	356	8.17E-10	5.43E-08
ECM-receptor interaction	mmu04512	22	87	0	0
Protein digestion and absorption	mmu04974	18	88	2.89E-15	2.56E-13
Amoebiasis	mmu05146	13	120	7.07E-08	2.69E-06
Hypertrophic cardiomyopathy (HCM)	mmu05410	12	86	1.28E-08	6.83E-07
Dilated cardiomyopathy	mmu05414	12	90	2.17E-08	9.63E-07
Cardiac muscle contraction	mmu04260	9	79	4.90E-06	0.000162783
Tight junction	mmu04530	8	138	0.001771256	0.049590264
Malaria	mmu05144	5	55	0.001864296	0.049590264
**P-PM VS P-LM**					
Focal adhesion	mmu04510	21	206	4.74E-11	4.21E-09
PI3K-Akt signaling pathway	mmu04151	20	356	2.85E-06	9.46E-05
ECM-receptor interaction	mmu04512	16	87	1.15E-12	3.06E-10
Protein digestion and absorption	mmu04974	15	88	1.86E-11	2.47E-09
Hypertrophic cardiomyopathy (HCM)	mmu05410	12	86	2.09E-08	1.39E-06
Complement and coagulation cascades	mmu04610	11	77	6.34E-08	3.37E-06
Dilated cardiomyopathy	mmu05414	11	90	3.25E-07	1.44E-05
Cardiac muscle contraction	mmu04260	10	79	8.04E-07	3.06E-05
Amoebiasis	mmu05146	10	120	3.53E-05	0.001042685
Tight junction	mmu04530	9	138	0.000546591	0.014539316
Thyroid cancer	mmu05216	4	29	0.001325109	0.032043544
**P-LM VS H-LM**					
Ribosome	mmu03010	10	161	1.22E-15	3.25E-13
**P-PM VS H-PM**					
Complement and coagulation cascades	mmu04610	10	77	3.50E-12	9.31E-10
Ribosome	mmu03010	6	161	0.000126348	0.016804306

## Discussion

Although some DEGs between skeletal muscles have recently been identified in several species, such as pigs [18, 19], mice [20], turkey [8], sheep [21], and dog [22], the molecular mechanism underlying muscle development in duck remains unclear. Here, we analyzed the DEGs between duck breeds with different growth rates using RNA-Seq technology. P and H specimens with the same embryo day and different growth rates were used as our study animals. In poultry, the form of secondary myofibers, which accounts for the majority of skeletal muscle fibers, happened from E8 to E16 stage [23]. Meanwhile, our phenotypic data shown that leg muscle weight: embryo weight ratio is only significantly different between H and P breeds at E15 (Fig 1D). Gu et al’s research indicated that from E13 to E19 is the fastest growth stage of Peking duck pectoral muscle in embryo [24]. Based on these acknowledge, we believe E15 is the proper day to investigate DEGs between locations and breeds. The goal of the current study was to identify global genes and pathways affecting duck skeletal muscle deposition between locations and breeds.

This study was specifically designed to identify DEGs between two duck breeds and two muscle locations within a breed rather than assessing differences between individuals. Therefore data analysis was performed on data collected from mixed sample pools comprised of tissues from 5 individuals. This design allowed us to analyze genetic data from 5 individuals of each duck breed at once, thereby providing us with a cost-effective way to minimize the possibility of identifying individual-specific DEGs rather than the desired breed-specific DEGs. Confirmation of the RNA-seq results with RT-PCR experiments provides greater confidence in DEGs identified. We acknowledge that this experimental design, while commonly employed, does not yield biological or technical replicates and all conclusions were drawn with this in mind.

### Analysis of library data

According to the muscle weight data, it is clear that dramatic phenotypic differences exist between LM and PM and between P and H. One phenotypic example is that PM has a higher degree of protein metabolism (high expression level of mTOR, S6K, FoxO1, MuRFbx and MAFbx), mainly in response to a higher growth rate, than LM in the Peking duck [14]. Any phenotypic differences may be associated with differentially expressed genes between the two locations or the two breeds. As a powerful tool, RNA-Seq was used to index these DEGs between LM and PM in both of the ducks. A total of 15 genes were randomly selected to verify the accuracy and repeatability of the sequence data using RT-PCR. Although all 15 genes have the same expression pattern between RT-PCR and DGE data, some genes have a different expression level between RT-PCR and DGE at a specific muscle tissue. This may be due to that the 2^-ΔΔCt^ analysis has less accurate than RNA-Seq technology, especially for lowly expressed gene. Compared with RT-PCR, the fold changes of these gene expressions were larger when measured by RNA-Seq. In addition, 4 of 15 genes were low-expressed genes, including FBLN5, PENK, RET and RSPO3 (0.76<RPKM<46.28), as verified using RT-PCR. This result is probably because the RNA-Seq was more sensitive in determining gene expression levels, particularly for low-abundance transcripts [25]; the same phenomenon has also been reported in other studies [21].

The identification of DEGs between leg muscle and pectoral muscle tissues from 2 different duck breeds indicates that location differences accounted for more of the genetic profile differences than did breed differences. This result also confirmed that different locations shared many more myogenetic genes and pathways than did different breeds. The functional annotation analysis also presented similar GO biological process terms and KEGG pathways between locations but not between breeds, whereas DEGs matched to only 1 GO biological process (“translation”) and 2 KEGG pathways (“ribosome” and “Complement and coagulation cascades”).

### Function of DEGs implicated in muscle development

Muscle fibers are the basic elements for muscle deposition. In different mammalian breeds, skeletal muscles contain four myosin heavy-chain (MyHC) isoforms, slow/β-, 2a-, 2b- and 2x-MyHC, and three major myosin light-chain (MLC) isoforms, the “slow” MLC1s and the “fast” MLC1f and MLC3f [26, 27]. Based on the expression of the four MyHC gene isoforms, muscle fibers are characterized into four different fiber types: I, IIA, IIB and IIX [28]. In this study, MYH7b was identified as a differentially expressed gene in Experiment 1 and 2. MYH15 was only identified in Experiment 1 (between LM and PM) and down-regulated in PM. On the other hand, three MYL genes (MYL1, MYL3 and MYL10) were found to be DEGs in Experiment 1, and MYL10 was also up-regulated in P-PM compared to H-PM. All of these variances in the gene profile can provide explanation for the differences in the fiber types in skeletal muscle for different locations and breeds. A previous study identified MYH3 and MYH8 as differentially expressed genes between intact and castrated cattle [29], indicating that these two genes are major genes for muscle fiber properties. We believe that these genes (MYH7b, MYH15, MYL1, MYL3 and MYL10) partially contribute to the difference in skeletal muscle deposition between locations and duck breeds.

The thin filament regulatory proteins troponin and tropomyosin are responsible for striated muscle contractions according to the effect of the intracellular Ca^2+^ concentration. Troponin consists of three subunits [30]: the Ca^2+^-binding troponin C (TNNC), the inhibitory troponin I (TNNI) and the tropomyosin-binding troponin T (TNNT), which interact strongly with each other. Previous studies have suggested that human mutations in TNNT3, TNNI2, and TPM2 increase the contractility of fast-twitch muscle fibers and cause distal arthrogryposis (DAs) disease [31, 32]. During myogenesis in vitro, Troponin I and slow MYBPC isoforms (MYBPC1) had a predominant expression in proliferating human mononucleated myoblasts and myotubes [33], and MYBPC1 is also a novel gene that is responsible for DA1 [34]. In the current study, these genes (TNNI2, TNNT3, TPM2/3 and MYBPC1) displayed the same profile, being down-regulated in PM. Another down-regulated gene in PM, FBN1, is a member of the homologous molecules family and regulates the structure and function of microfibers and elastic fibers, which provide an extracellular reservoir for inactive growth factor [35]. Mutations in FBN1 cause an autosomal dominant connective tissue disorder, Marfan syndrome (MFS), which displays variable manifestations in the skeletal, cardiovascular and ocular systems [36, 37]. As the only protein that has been unambiguously implicated in determining limb-type morphologies, Pitx1 is necessary for the normal initiation of hind limb outgrowth as a result of the regulation of Tbx4 expression [38]. Tbx4, which is exclusively expressed in the hind limb, plays a crucial role in hind limb bud initiation [39]. Those genes that were down-regulated in PM indicate that duck LM had a higher growth rate than PM at embryonic day 15, indicating that the leg muscle has an earlier developmental origin than does the pectoral muscle. CTNNB1, which is a primary mediator of the WNT/β-catenin signaling pathway, is responsible for skeletal myogenesis involving hypertrophy [40, 41]. A previous study demonstrated that the CTNNB1 gene had an increased expression in Pietrain fetuses compared to that in Duroc fetuses [42], but this gene expression as detected by qRT-PCR did not confirm the results observed with the microarray. In the present study, we found only CTNNB1 to have a location-special expression pattern. For its breed-specific expression pattern, further studies may be warranted.

### Function of some pathways related to muscle development

Based on the functional annotation analysis of DEGs from different muscle tissues, we identified the predominant differentially expressed genes as being related to focal adhesion, the PI3K-Akt signaling pathway and ECM-receptor interactions for location-specific DEGs in Experiment 1 and as being related to ribosomes for breed-specific DEGs in Experiment 2.

Focal adhesions are integrin-based structures that determine the adhesive behavior of cells in response to cell migration, growth, and differentiation [43, 44]. Fibronectin (FN) and vitronectin (VN) are two common and vital components. We found that FN and VN are location-specific genes and that VN is also a breed-specific gene in PM. Burridge’s review (1988) mentioned that in avian cells, integrin is concentrated even within those focal adhesions lacking FN and indicated that VN is probably another ECM component that binds to integrin [43]. In this study, we found that FN1 and VN had contrasting expression patterns in Experiment 1 for Peking ducks, indicating that their role in skeletal muscle growth warrants further study. The study by Timmons et al (2005) demonstrated that FN1 increased the expression of the Laminin gene family (LAMA4, LAMB1, and LAMC1) by endurance exercise training [45]. LAMA2 and LAMC1 play distinct roles in myogenesis [46]. Mutation in the LAMA2 gene causes merosin-deficient congenital muscular dystrophy (MDC1A) [47]. The differential expression of LAMB1 has been observed during pig muscle development [48] [49], and LAMB1 is a positive factor in the activation of myofiber formation [49]. These DGEs displayed a different expression pattern in Experiment 1, suggesting that the focal adhesion pathway contributes to the difference in skeletal muscle development between locations and that these DGEs could be the main candidate gene for this difference.

PI3K is pivotal in growth factor-, insulin- and G protein-mediated signal transduction and is involved in adhesion and migration regulation. In addition, the PI3K/AKT pathway plays an important role in the process of myotube differentiation [50, 51], which is mediated by the dystrophin-glycoprotein complex (DGC). The disruption of DGC induced apoptosis in muscle cell cultures by decreasinged phosphorylation of AKT and its downstream effector GSK-3β [52]. The inhibition of PI3K by specific inhibitors reduces adhesion and migration in a variety of cell types [53–55]. Down-regulated Akt activation by overexpressed SHIP-2 causes cell-cycle arrest [56]. In addition, many studies have demonstrated that the AKT/mTOR (mammalian target of rapamycin) signaling pathway is activated during hypertrophy [57] and improves the increase in muscle mass according to the increase in muscle fiber size [58]. Although we did not detect PI3K and AKT as DEGs between locations and breeds, some genes involved in the PI3K/AKT pathway caught our attention. Down-regulated LAMA2, LAMC1, TNNI2 and TNNT3 and up-regulated angiopoietin 2 (ANGPT2) and PDGFC/D are enriched in the PI3K/AKT pathway. ANGPT2 expression increases significantly during myoblast differentiation into myotubes and promotes skeletal myoblast survival and differentiation though the activation of the PI3K/AKT and Erk1/2 pathways [59]. The PDGF family consists of four members: PDGF-A, PDGF-B, PDGF-C and PDGF-D, and the last two members (PDGF-C and PDGF-D) were recently discovered and were found to be DEGs in Experiment 1 in the present study. The review by Reigstad (2005) et al showed that both PDGF-C and PDGF-D are involved in various malfunctions: PDGF-C seems to play an important role in Ewing family sarcomas, whereas PDGF-D is related to lung, prostate and ovarian cancers [60]. Until now, information about the roles of PDGF-C and PDGF-D in skeletal muscle development has been limited; our finding that PDGF-C and PDGF-D are location-specific genes in skeletal muscle indicates that these two genes may play a special role in skeletal muscle development and require further study.

ECM-receptor interactions play a profound role in major cellular programs, including migration, growth, difference and survival [61], and play an important role in myogenesis [62]. Collagen (types I, III, IV, and V) [63, 64] and fibronectin [65] are the main constituents of ECM in skeletal muscle tissue. Collagen type V is probably already suggested to be involved in the sequence of events leading to myoblast differentiation [64]. In this study, a total of six collagen members (COL11A1, COL12A1, COL14A1, COL1A2, COL5A2 and COL9A1) were differently expressed in Experiment 1 and Experiment 2. Previous studies have also reported that many other collagen family numbers have different expression patterns during skeletal muscle development [8, 19]. Taken together, these findings indicate that collagen gene expression variability plays an important role in affecting muscle development.

Interestingly, some pathways in Experiment 1 are highly related to cardiac development, including the hypertrophic cardiomyopathy (HCM), dilated cardiomyopathy and cardiac muscle contraction pathways, which indicates that these DGEs in skeletal muscle also have a link with cardiac development. This is consistent with previous studies wherein genetic manipulation conducted for many years resulted in increased muscle production while promoting dysfunction of cardiac development [66]. However, the mechanism linking muscle development and cardiac disease remains incomplete and requires further study. Yet another difference we identified between LM and PM is that protein digestion and absorption pathway is also significant between locations, demonstrating that leg and pectoral muscle already display differential protein metabolism during the embryo stage. This variation in protein metabolism could, conceivably, be the main reason for muscle mass gain difference after post-hatching [14].

Breed-specific DEGs identified in Experiment 2 are all pertaining to ribosome assembly and function. This is interesting, as ribosomes are platforms upon which to perform protein synthesis and thereby promote skeletal muscle deposition. Many ribosomal proteins (RPS6, RPS23, RPL36, RPSA, RPL8, etc.) and Elongation factor 1 (eEF1) (eEF1A1, eEEF1A2 and eEF1D) were detected by RNA-Seq in the present study, especially in Experiment 2. eEF1 is responsible for transferring aminoacyl-tRNA to the empty A-site on the ribosome [67]. In line with a previous study reporting that protein metabolism is the main reason for promoting duck skeletal muscle deposition [14], these findings indicate that protein synthesis may also be the main determinant for differences in skeletal muscle development between duck breeds.

## Conclusions

Taken together, all of the data from the present study demonstrate obvious differences in muscle weight and gene expression between leg and pectoral muscles and between Heiwu duck and Peking duck. It is intriguing that were more location-specific genes identified than breed-specific genes. The GO results suggested that location-specific functional gene groups are conserved between two duck breeds, and include a much broader range of functions than do the breed-specific functional groups. This study suggests that focal adhesion, the PI3K-Akt signaling pathway and ECM-receptor interaction may be the main molecular networks that are responsible for muscle development differences between leg and pectoral muscles. Furthermore, our findings suggest that ribosomes could be the main molecular driver of the differences between the two duck breeds. Thus, this study successfully identified candidate genes and pathways involved in the differences in muscle growth between leg and pectoral muscles and between Heiwu duck and Peking duck, and it might provide the basis for future experiments that focus on these candidate genes, their proteins products and their functions in duck skeletal muscle.

## Supporting Information

S1 Excel List of differentially expressed genes in each library comparison. DEGs between pairs of libraries are shown (H-PM vs H-LM, P-PM vs P-LM, P-LM vs H-LM and P-PM vs H-PM).
(XLSX)

S1 Fig Cluster analysis of differentially expressed genes according to Pearson’s correlation coefficient. (TIF)

S1 Table The primer information for RT-PCR. (DOCX)
