# Supplementary figures and images for: Transcriptional Profiling Identifies Location-Specific and Breed-Specific Differentially Expressed Genes in Embryonic Myogenesis in Anas Platyrhynchos
Source: PLoS One. 2015 Dec 2;10(12):e0143378. doi: 10.1371/journal.pone.0143378 (PMC4667915; doi:10.1371/journal.pone.0143378)

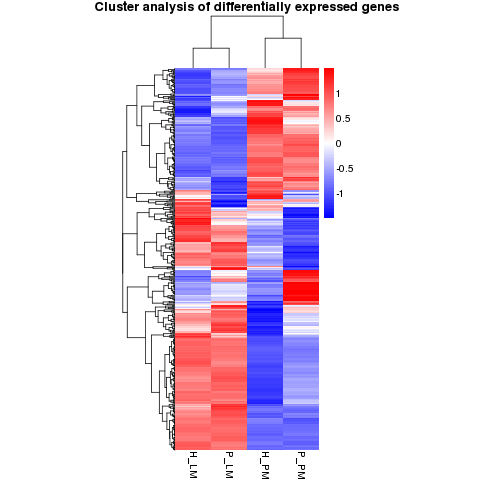

Supplement: S1 Fig — (TIF) [file pone.0143378.s002.tif]
