# Supplementary material for: Transcriptional Profiling Identifies Location-Specific and Breed-Specific Differentially Expressed Genes in Embryonic Myogenesis in Anas Platyrhynchos
Source: PLoS One. 2015 Dec 2;10(12):e0143378. doi: 10.1371/journal.pone.0143378 (PMC4667915; doi:10.1371/journal.pone.0143378)
Supplement: S1 Table — (DOCX) [file pone.0143378.s003.docx]

Table S1 the primer information for RT-PCR

| **Gene Name** | **Primer Sequence** | **Temperature (℃)** | **Production length (bp)** | **NCBI Reference Sequence Number** |
| --- | --- | --- | --- | --- |
| ACTA1 | F: TGACTGAGCGTGGCTATTC  R: CCCATCAGGCAACTCGTA | 53.8 | 158 | XM_005020223.1 |
| ACTC1 | F: TCACCAACTGGGACGAT  R: GCATAGAGGGACAGGACA | 51.3 | 205 | XM_005009784.1 |
| ANGPT2 | F: GACCGTTCATAACTTGC  R: ATTGTCCAGCCTCCTC | 47.6 | 210 | XM_005009208.1 |
| APOBEC2 | F: GAGACCCAAGGCAAAGA  R: AGGGACTGGAGGAGACG | 51.1 | 154 | XM_005026827.1 |
| EEF1A1 | F: TTCCACCGAGCCACCTT  R: GGTAACCTTCCATCCCTTG | 51.6 | 184 | XM_005009711.1 |
| ENO1 | F: GCGATGGAAAGTATGACC  R: CCAACGCTGCCAGTAAA | 52.2 | 178 | XM_005024236.1 |
| FBLN5 | F: GCAACCCAGGCTTTACC  R: CTCCGTCAGCTTCCAGT | 50.9 | 153 | XM_005021441.1 |
| LOC101792412 | F: ATGGCAAGAAGGTGGCG  R: AGCAGGGTGGTGGATGG | 55.6 | 173 | XM_005031463.1 |
| HOXA6 | F: GGCGGGCTATGATGCT  R: CAAGGACGGTGTTGGACT | 52.4 | 110 | XM_005022081.1 |
| LAMB2 | F: CAGGAGACGGAGGACTTAG  R: TTAGGGTCGCCAGTTGA | 52.0 | 149 | XM_005009838.1 |
| PENK | F: CGGGTAGAACCAGAGGAT  R: CAGCAGGTCAGAGGAATT | 50.3 | 114 | XM_005012125.1 |
| RET | F: TATGTGGGTAGTGATGGTA  R: TTTCTGGCTGCTAAATC | 46.8 | 161 | XM_005015789.1 |
| RPSS35 | F: TTGCCTACATTGGGTCT  R: CAGCTCTAAGTTGGGTT | 45.9 | 141 | XM_005024797.1 |
| RSPO3 | F: TACGGCGACAGGGAAGA  R: GGACACGAGGAAAGACATAC | 51.5 | 163 | XM_005009596.1 |
| SEMA3C | F: GCAGGCAAGATGTGAGA  R: CTTGGACAGAGCGAATG | 50.1 | 252 | XM_005016705.1 |
